# Inflammation promotes erythropoietin induced vascular calcification by activating p38 pathway

**DOI:** 10.1080/21655979.2022.2038430

**Published:** 2022-02-15

**Authors:** Xunjia Li, Chengxuan Liu, Ying Li, Weijian Xiong, Deyu Zuo

**Affiliations:** a Department of Nephrology, Chongqing Hospital of Traditional Chinese Medicine, Chongqing, China; b Department of Rehabilitation Medicine, Chongqing Hospital of Traditional Chinese Medicine, Chongqing, China

**Keywords:** Erythropoietin, inflammation, vascular smooth muscle cells, vascular calcification

## Abstract

The current research aimed to verify the effects of erythropoietin (EPO) on vascular calcification under inflammatory conditions and the molecular regulator of vascular calcification induced by EPO. To induce vascular calcification and systemic chronic inflammation in SD rats, EPO was administered intraperitoneally, and 10% casein was injected subcutaneously. The administration period lasted for 20 consecutive weeks. Blood samples were subsequently collected to detect inflammatory factors and vascular calcification. Additionally, high-dose EPOs were applied to stimulate primary vascular smooth muscle cells (VSMCs), and vascular calcification was measured using alizarin red staining, alkaline phosphatase (ALP) activity, and calcium salt quantification. The probe 2’,7’-dichlorofluorescein diacetate (DCFH-DA) was employed to detect cellular reactive oxygen species (ROS) levels. The expressions of bone formation-related protein and anti-calcification protein matrix gla protein (MGP) were determined via Western blot. Compared with the control group, calcium deposits and vascular calcification were increased in the EPO group, tumor necrosis factor-alpha (TNF-α) group and TNF-α+ EPO group, whereas MGP was significantly reduced. Moreover, under the stimulation of TNF-α and EPO+TNF-α, pp38/p38 was increased substantially, the addition of p38 inhibitor SB203580 could significantly reduce calcium deposits and vascular calcification. In vivo experiment, compared with the EPO group, calcium salt deposition and vascular calcification were elevated in the EPO+casein group. The present results revealed that high-dose EPO could cause calcification of the abdominal aorta in rats. The inflammatory response aggravated the vascular calcification induced by EPO via activating p38 and ROS levels.

## Graphical Abstract

**Figure:**
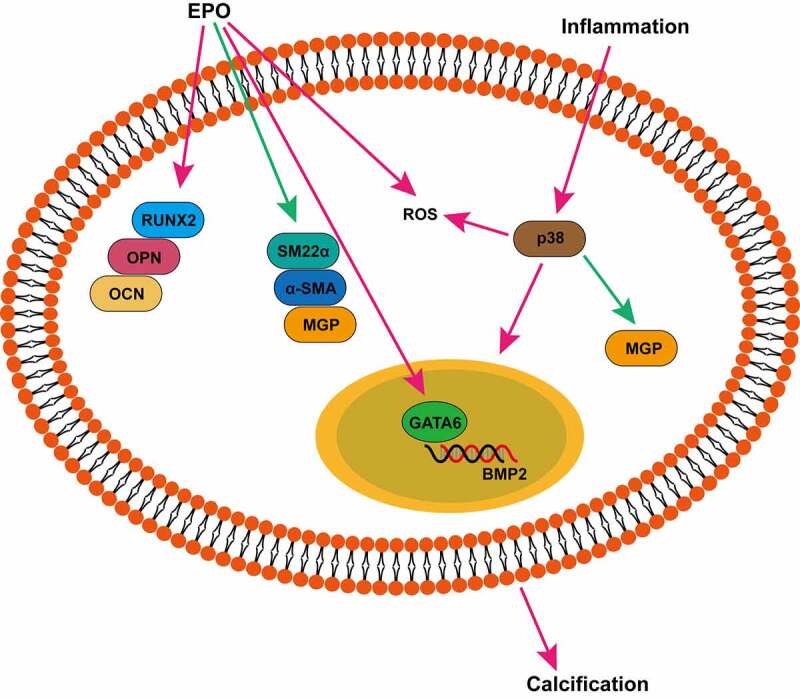

## Introduction

Vascular calcification is a known commonplace pathology of senescence, atherosclerosis, diabetes, and hypertension, and it corresponds to increased risks of vessel stiffening and myocardial infarction [1]. The most catastrophic manifestations occur in patients with chronic kidney disease (CKD) who suffer from substantially increased risks of cardiovascular death versus the age-matched control group [1]. When CKD patients accompanied by calcification, the risk of development of cardiovascular disease is 1.23–2.70 times as high as those spared from abdominal aorta calcification [2–5]. It is currently considered that vascular calcification is an active process. The phenotypic transformation, abnormal calcium and phosphorus balance, vascular smooth muscle cells (VSMCs) apoptosis, oxidative stress, aberrantly degraded smooth muscle elastic membranes, and damaged anti-calcification mechanisms of VSMCs are all involved in calcification [6–12]. Median calcification occurs in the smooth muscle layer of the large and medium arteries [13]. The pathological manifestation is the deposition of calcium salts in the inner elastic layer of the artery, leading to an increase in vascular stiffness, systolic blood pressure, pulse pressure, and pulse rate. Consequently, most CKD patients suffer from serious consequences of diastolic dysfunction, left ventricular hypertrophy, and heart failure [14].

Erythropoietin (EPO) has emerged as an efficient agent for renal anemia in CKD. However, some research on CKD patients has reported that high-dose EPO links to cardiovascular complications and all-cause mortality, and weekly administration of EPO has been regarded as a cardiovascular event risk factor [6–9]. Numerous studies have pointed out that EPO works directly on VSMCs and affect cell survival, proliferation, and functional status [10–12]. Influences of EPO pro-calcification have also been demonstrated in our previous studies [15,16]. We found that EPO could induce calcification in a dose-dependent manner and it elevated expressions of runt-related transcription factor 2, osteopontin, and osteocalcin genes associated with bone formation and alkaline phosphatase (ALP) activity in VSMCs. Besides, EPO upregulated transcription factor GATA Binding Protein 6 (GATA6), which binds to the promoter of bone morphogenetic protein-2 (BMP2) [17], a key protein in calcification, and led to calcium deposition in VSMCs [15]. In vivo, EPO could not only induce vascular calcification in normal rats but also aggravate it in CKD rats, yet the mechanism remains unclear.

CKD is believed to be a chronic, low-grade inflammatory condition, characterized by elevated levels of interleukin (IL)-6, C-reactive protein (CRP), innate immunity biomarkers, as well as TNF-ɑ [18–21]. There are several causes for the microinflammation status in CKD, namely uremic milieu, infection, oxidative stress, and intestine microbiota [22]. Microinflammation in CKD accelerates the advancement of chronic inflammatory disorder mediating immune balance [23]. Both inflammation and oxidative stress signal transduction have been regarded as the primary factors in the pathogenesis of vascular mineral deposition [24], implying that inflammation may be essential in the pathogenesis of CKD vascular calcification.

It is assumed that inflammation can exacerbate EPO-induced vascular calcification. Thus, we have designed experiments to investigate the effect of high-dose EPO on vascular calcification in a state of microinflammation in vitro and in vivo. Though high-dose EPO causing VSMC calcification has been proved via experiments in vitro, its mechanism is still unclear. In addition, an existing state of microinflammation has been found in CKD patients acting as a risk factor of vascular calcification. It is unclear whether inflammation can play a superimposed or synergistic effect in EPO-induced vascular calcification. Collectively, this study was carried out to explore the role of EPO driving vascular calcification and its molecular regulation mechanism in an inflammatory state. Meanwhile, it was also expected to provide strategies for clinical prevention and control of vascular calcification caused by high-doses EPO in CKD patients.

## Methods and materials

In this study, EPO and TNF-α were used to stimulate primary vascular epithelial cells of rats. SD rats were fed in a high-phosphorus diet and injected with EPO intraperitoneally and with casein subcutaneously to construct a model of vascular calcification. Combined experiments in vitro and in vivo, a series of experiments including alizarin red staining, ELISA, ROS detection, ALP activity detection, qPCR, Western blot, and drug intervention were used to explore the molecular mechanism of EPO-induced vascular calcification and to preliminarily investigate the EPO inflammatory state on vascular calcification and related signal transduction mechanisms. Our previous studies have revealed that high-dose EPO can promote the calcification of VSMCs [16], and CKD patients are under a long-term microinflammatory state. Furthermore, inflammation is also a risk factor for vascular calcification, and we speculate that inflammation can promote EPO-induced vascular calcification.

### Animal experiments

The animal experiments described in the present work were all gained approval of the Animal Ethics Committee of Chongqing Medical University and the Animal Management Rule of the Chinese Ministry of Health. Eight-week-old male SD rats were bought from the Animal Experimental Center of Chongqing Medical University. All rats were provided with high phosphorus (1.2%) diets under conditions of 12-h light–dark cycles at 23°C. Followed one-week adaptive feeding, the laboratory animals were classified into four groups randomly (10 of each): control group, EPO group, casein group, and EPO+casein group. High-dose EPO 2000 U/Kg/W (Life-iLab, Shanghai, China) was employed for injections intraperitoneally to rats in both EPO and EPO+casein groups three times per week, lasting for 20 consecutive weeks. Simultaneously, 10% casein (1.2 g/kg) (Sigma, USA) was subcutaneously injected into the rats in casein group and the EPO+casein group every other day, also lasting for 20 weeks [25]. The control group was administered normal saline (Southwest Pharmaceutical Co., Ltd.) at an identical quantity. Ultimately, all rats were put to death to collect the abdominal aortic vessels for subsequent experiments.

### Primary VSMCs culture

The healthy animals purchased from the Animal Experimental Center of Chongqing Medical University were anesthetized with high dose chloral hydrate, sterilized by being immersed in 75% alcohol for 1 min, and followed by irradiation with ultraviolet on a sterile bench for 30 min. Rat thoracic aortas were obtained, cleaned twice using PBS (Solarbio, China), prepared into small pieces using ophthalmic scissors, inhaled into a sterile tube and placed in a culture flask adherent to the wall. The samples were incubated under the condition of 5% CO_2_ for 1 h and placed horizontally. Of 3 mL high glucose DMEM (Gibco, USA) which contained 20% fetal bovine serum (Gibco, USA) was added [26]. The cells were kept for 1 week in the incubator and observed under a microscope as VSMC cell growth appeared from tissue segments with a shape of typical spindle.

### Cell administration

VSMCs were cultured normally in an incubator (Thermo, USA) under the condition of 5% CO_2_, and experiments were started when cells had grown to around 80% confluence. The normal cultured cells were used as the control group. The TNF-α+ EPO group was treated with EPO (250 U/L) and TNF-α (20 ng/mL) for 14 d. The medium was changed every 2 days and EPO and TNF-α treatment was given after the medium was changed. The group treated with TNF-α or EPO alone was also treated with TNF-α (20 ng/mL) and EPO (250 U/L) for 14 d, respectively. In addition, in p38 inhibitor SB203580 and ROS scavenger pretreatment group, VSMCs were pretreated with SB203580 (10 μmol/L) and NAC (150 μmol/L) for 1 h, and then EPO or TNF-α was added. TNF-α and SB203580 were from Sigma (USA). NAC was purchased from Beyotime, China.

### Calcium deposition detection in the abdominal aorta and extracellular matrix using alizarin red staining

Calcium deposition detection was performed according to the manufacturers’ instructions (Solarbio, China) [27]. Briefly, VSMCs were placed in a 6-well plate at a density of 2 × 10^5^ cells/well. The cells were grouped as above, and the cells were serum starved for 1 d, and then treated for 14 d according to the grouping conditions. Then, following PBS washing, 4% paraformaldehyde (Solarbio, China) was employed for VSMCs fixation for 15 min. The cells were stained for 30 min in the dark by adding Alizarin red S dye solution (Solarbio, China) and visualized applying a fluorescence microscope (ZEISS, Germany) through which calcium salts displayed in red crystals.

### Serum inflammatory factor detection by enzyme linked immunosorbent assay (ELISA)

Blood samples were allowed to coagulate naturally at room temperature for 20 min, and then centrifuged at 3 000 r/min for 15 min. Supernatants from blood samples of SD rats were used to analyze levels of IL-1β, IL-6, TNF-α and IL-10 by ELISA as per manufacturers’ instructions (Uscn Life Science, Inc.). The optical density (OD) was detected in 15 min at 405 nm on a microplate reader (Thermo, USA).

### Reactive oxygen species (ROS) detection in VSMCs

We then performed experiments to detect the intracellular ROS generation applying a 2’,7’-dichlorofluorescein diacetate (DCFH-DA) fluorescent probe (Sigma, USA) [28]. Cells were seeded in 6-well plates at 2 × 10^5^ cells/well and treated with single EPO (250 U/mL) or combined with TNF-α (20 ng/mL) in the presence or absence of p38 inhibitor SB203580 (10 μmol/L) for pretreatment 8 d. The cells were cultured at 37°C for 30 min using10 μmol/L DCFH-DA, rinsed with PBS, and visualized via fluorescence microscopy. The excitation wavelength and emission wavelength were at 488 and 525 nm, respectively.

### Bicinchoninic acid (BCA) assay detection of protein concentrations

Drug intervention was performed first and followed by protein extraction. Standard protein diluent (Solarbio, China) and BCA solution (Solarbio, China) were prepared as per BCA instructions. Using 96-well plates, the standard protein diluent and samples of each group were supplied and the total volume of each well was 20 μL. Subsequently, each well of the plates was supplemented with BCA working solution 200 μL and incubated at 37°C for 30 min. The well absorbance at A562 was determined using an enzyme reader and a standard curve of protein concentrations was plotted using EXCEL.

### Cellular calcium quantitative detection

The samples were intervened with drug and the culture medium was removed. Followed by three cycles of PBS washing, 2 mL, 0.6 mmol/L of hydrochloric acid (HCL) (Sangon Biotech, China) was supplied to each hole of 6-well plates for incubation 24 h at room temperature, collected the supernatant, and determined calcium concentration at 24 h. The calcium concentration was detected by QuantiChrom^TM^ Calcium Assay Kit according to the manufacturers’ instructions (QuantiChrom, USA). Of 5 μL sample was added to 96-well plates, supplemented with 200 μL of working solution, and vibrated at room temperature for 3 min. The absorbance at 612 nm was measured using a CMax Plus microplate reader (Thermo, USA). Based on the obtained concentration, we calculated the total amount of calcium ions in the supernatant of all groups. The formula used for calculation was presented: Calcium ions (μg) = Calcium ion concentration in the supernatant (mmol/L) × 40 × 10^3^ μg/L × 2 × 10^−3^ L. Total protein contents of each group were also calculated using the BCA method. Calcium ion contents per milligram of protein in each group was obtained when the total amount of calcium ions in each group was divided by total protein content.

### ALP activity determination

Cells were seeded in 6-well plates at 2 × 10^5^ cells/well and treated with drugs as previously described. ALP activity was determined by Alkaline Phosphatase Activity Assay Kit (Nanjing Jiancheng Technology Co., Ltd.). Each well was added with 150 μL RIPA lysate (Beyotime, China) at 4°C, and full rupture of cells was required by being blown repeatedly using a straw. The supernatant was obtained after centrifugation (4°C, 15,000 r/min) for 15 min and used for ALP activity detection as manufacturers’ instructions. Each well was added with 5 μL sample and 100 μL substrate which contained p-nitrophenyl ester disodium (Sigma, USA). The reaction was terminated by being supplemented with 1 mol/L NaOH 30 min later, and detection was performed at A520 nm using an enzyme labeling device (Thermo, USA).

### Western blot assay

The cells were collected using a scraper, mixed evenly, dissolved on ice using lysate for 40 min, placed into a pre-cooled EP tube and centrifuged at 4°C 14000 rpm for 15 min. Protein concentration was detected using BCA kit (Life-iLab, Shanghai, China). Following electrophoresis at 100 V for 40 min and 80 V for 1 h, membrane transference was carried out at 250 mA and lasted for 2 h. The membrane blocking was performed using 5% skimmed milk at room temperature for 1 h, followed by addition of primary antibodies, vibrated for 1 h at room temperature. The membrane followed three cycles of washing using Tris-buffered saline with Tween 20 (TBST), 10 min each time. Secondary antibodies were subsequently supplemented for 1 h incubation at room temperature and rinsed twice using TBST [29]. Photographs were taken for grayscale analysis via Image J software. The antibodies were listed as following: BMP2 at 1:2000 rate (BM0231, IGEE), GAPDH at 1:1000 rate (5174S, CST), GATA6 at 1:1000 rate (BM16634, IGEE), MGP at 1:1000 rate (10,734-1-AP, PROTEINTECH), pp38 at 1:1000 rate (4511 T, CST), p38 at 1:1000 rate (8690S, CST), HPR Goat anti-rabbit IgG at 1:2000 rate (BMS041, IGEE) and goat anti-mouse IgG at 1:8000 rate (GAM007, MultiSciences).

### qPCR assay

Sample RNA was extracted and reversely transcribed as per instructions of reverse transcription kit (Promega, USA). Firstly, reverse transcription reaction was carried out using 4 μL RNA as a template and the loading machine was ABI9700 (ABI, USA). The reaction solution included: 4 μL 5 × RT buffer, 0.4 μL random primers, 1 μL MMLV (U/μL), 0.5 μL 10 mM dNTPs, 10.1 μL diethylpyrocarbonate (DEPC)-treated water (Sangon Biotech, China), and 4 μL RNA template. The conditions set for reaction were 37°C for 1 h and 95°C for 3 min. The reaction system included the following: PCR mix buffer 2 × 10 μL, 1 μL upstream primer and 1 μL downstream primer, 4 μL of template, and 6 μL of DEPC- treated water. The reaction was carried out at 93°C for 3 min, 93°C for 30 s, 55°C for 45 sec, with a total of 40 cycles, then 72°C for 5 min, and 4°C forever. The applied primers included: GATA6-F, 5’- GGATTCTTGGTGTGCTCTGG-3’; GATA6-R, 5’-ATTTTTGCTGCCATCTGGAC-3’; BMP2-F, 5’-TCTTCCGGGAACAGATACAGG-3’; BMP2-R, 5’-TCTCCTCTAAATGGGCCACTT-5’; MGP-F, 5’- TGAAGAGCCTGATCCTTCTTGCCA-3’; MGP-R, 5’- TAGAGCGTTCTCGGATCCTCTCTT-3’; β-actin-F, 5’-GATGCTCCCCGGGCTGTATT-3’; and β-actin-R, 5’-GGGGTA CTTCAGGGTCAGGA-3’.

### Immunofluorescence staining

The 4-μm-thickness paraffin sections were placed at 60°C for 2 h, dewaxed using xylene and hydrated with alcohol. Following the addition of citrate buffer (pH 6.0) repaired by antigen, the slices were placed in an oven, and heated for 20 min. They were subsequently cooled naturally to room temperature followed by three cycles of washing with PBS, each time for 5 min, blocked 30 min by being supplemented with 5% BSA. Primary antibodies (GATA6 at 1:1000, BM16634, IGEE; and BMP2 at 1:2000, BM0231, IGEE) were incubated overnight at 4°C. After three washes using PBS, 5 min each time, the sections were rewarmed 30 min. Goat anti-mouse IgG antibody (Alexa Fluor 488, 1:400, Invitrogen, USA) or donkey anti-rabbit IgG (Alexa Fluor 546, 1:400, Invitrogen, A10040, USA) was added for incubation 1 h at room temperature, followed by PBS washing 3 times, 5 min each time, incubated for 5–10 min using DAPI (Sangon Biotech, China) away from the light, and washed three times with PBS, 1 min each time [30]. Observations and photographs were performed via a fluorescence microscope (ZEISS, Germany).

### Statistical Methods

All data measured were analyzed using Graphpad Prism 6.0 software and presented by mean ± SD. Normality and homogeneity of variance of each group were tested. Multiple comparison among groups was performed using one-way analysis of variance with post hoc testing using the Tukey method. Values of *p* < 0.05 were considered statistically significant.

## Results

### TNF-α can aggravate EPO-induced calcification in VSMCs

**Figure 1.**
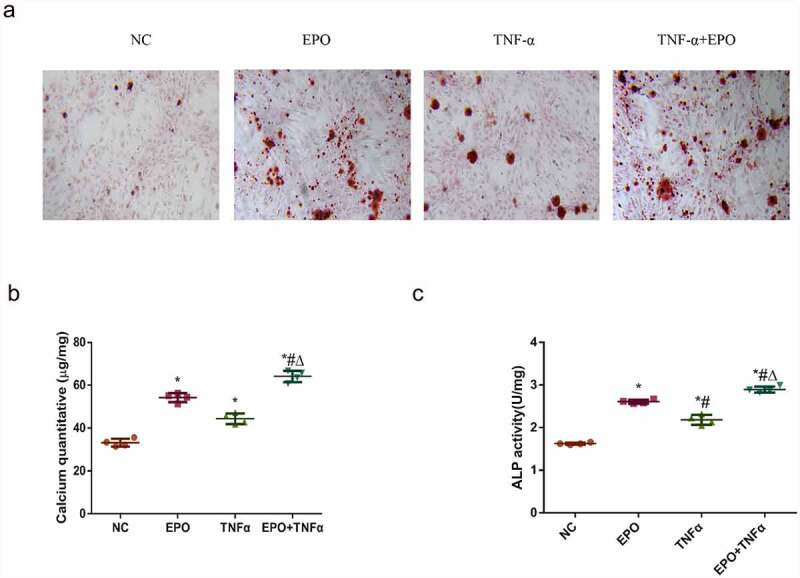
TNF-α aggravated EPO-induced calcification in VSMCs. VSMCs were cultured with single EPO (250 U/mL) or combined with 20 ng/mL TNF-α for 14 d. A. Alizarin red staining (magnification ×200). B. Determination of calcium content. C. Detection of ALP activity in VSMCs following 8 d treatment. The results are expressed as mean ± SD; *n*=3. * p<0.05 vs. NC group, # p<0.05 vs. EPO group, Δ p<0.05 vs. TNF-α group.

To determine whether inflammation could aggravate VSMCs calcification induced by EPO, VSMCs were treated with EPO (250 U/L) and EPO + TNF-α (20 ng/mL) for 14 d, respectively. Alizarin red staining and calcium quantification of VSMCs exhibited a moderate calcium deposition in the EPO group and the TNF-α group. Moreover, a significant increase of calcium deposition was revealed in the EPO + TNF-α group compared with the single EPO group (Figure 1(a,b)), indicating the acceleration of calcification by TNF-α treatment. We further examined the ALP activity of VSMCs after 8 d interference. ALP activity was markedly increased in the EPO + TNF-α group with the highest ALP activity (Figure 1(c)). These data indicated that TNF-α significantly potentiated EPO-induced calcification and ALP activity of VSMCs.

### Effects of EPO and TNF-α on osteogenic gene and anti-calcification protein expression

**Figure 2.**
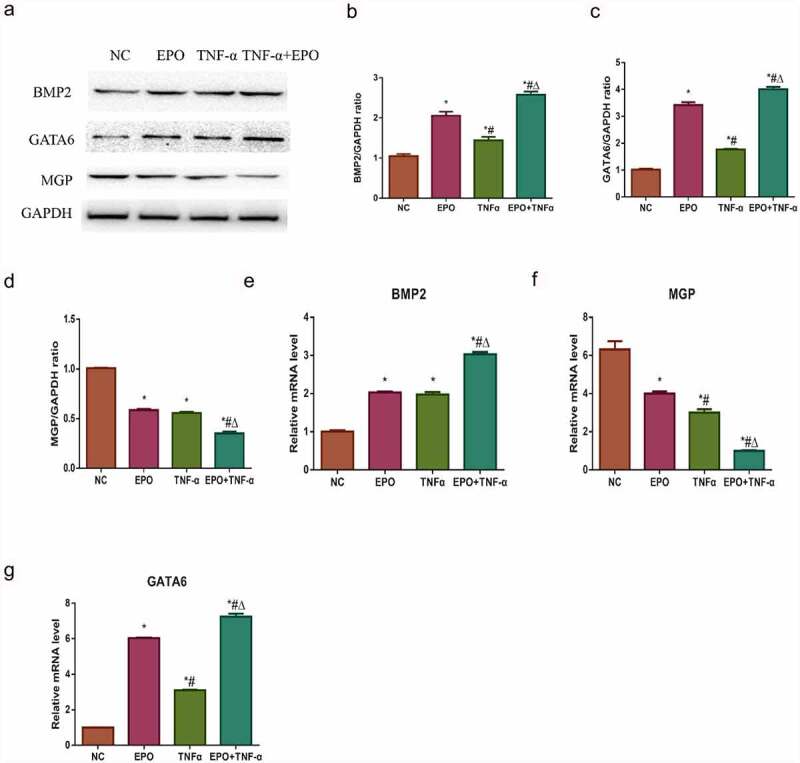
Effects of EPO and TNF-α on osteogenic gene and anti-calcification protein expression. VSMCs were cultured with single 250 U/mL EPO or combined with 20 ng/mL TNF-α for 8 d. A. Western blot results of BMP2, GATA6 and MGP protein expressions. B-D. Densitometric analysis of protein expression. E-G. The mRNA expressions of GATA6, BMP2 and MGP. The results are expressed as mean ± SD; *n*=3. * p<0.05 vs. NC group, # p<0.05 vs. EPO group, Δ p<0.05 vs. TNF-α group.

Alizarin red staining result indicated that TNF-α could aggravate EPO-induced calcification and ALP activities in VSMCs. In our previous study, we explored that EPO-induced calcification in VSMCs through GATA6/BMP2 axis [15]. Western blot and qPCR were used to study the possible molecular mechanism of TNF-α in exacerbating the EPO-induced calcification and to confirm the effects of TNF-α on GATA6 and BMP2. It was confirmed that both the gene and the protein expressions of GATA6 and BMP2 were significantly increased by TNF-α at day 8 in comparison with those in the control group. Besides, a marked increase was observed in the EPO + TNF-α group compared with the EPO group (Figure 2(a–c, e, and g)). Both EPO and TNF-α involved in decreasing MGP in mRNA and protein levels versus control, and a substantial decrease exhibited in the EPO+TNF-α group in comparison with both the EPO group and the TNF-α group (Figure 2(a, d and f)). These data suggested that TNF-α aggravated EPO-induced calcification by further upregulating GATA6 and BMP2 along with downregulating MGP.

### ELISA validation of chronic inflammation modeling

**Figure 3.**
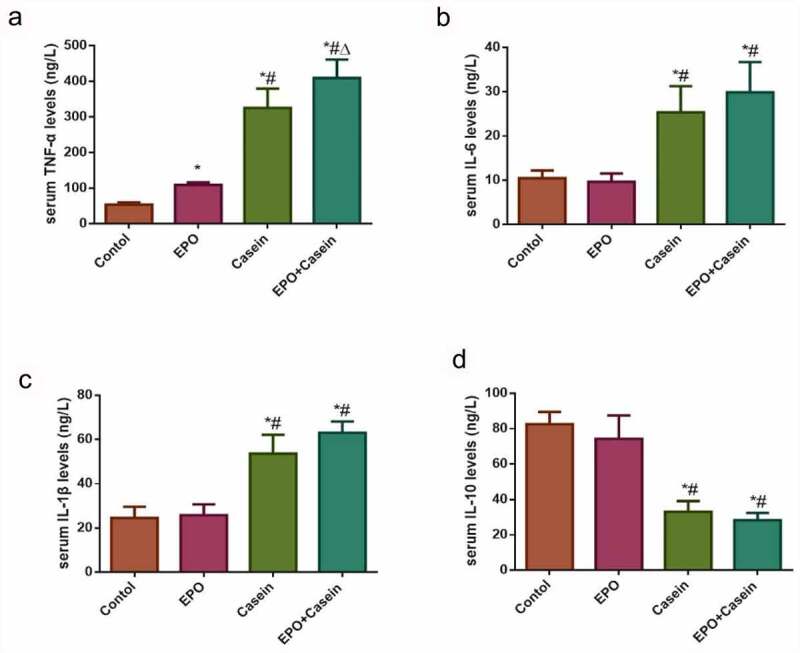
Levels of inflammatory factors in the serum of rats in each group. Rats were injected intraperitoneally with 2000 U/Kg/W of EPO three times per week or subcutaneously with 10% casein (1.2 g/kg), lasting 20 weeks. (a-d) The levels of TNF-α, IL-6, IL-1β and IL-10 in serum were detected using ELISA kits. The results were expressed as mean ± SD; *n*=5. *p<0.05 vs. Control group, # p<0.05 vs. EPO group, Δ p<0.05 vs. Casein group.

To verify whether the model of chronic inflammation was successfully constructed or not, the ELISA assay was conducted to detect levels of inflammatory factors in the serum of rats as shown in Figure 3. In contrast to the control group, the serum concentrations of TNF-α, IL-6, and IL-1β in the casein group and the EPO+casein group were increased significantly, whereas decreased in IL-10, while the serum TNF-α of the EPO group was also slightly elevated. The results proved that the model of chronic inflammation was successfully established.

### Chronic inflammation can aggravate vascular calcification caused by EPO in vivo

**Figure 4.**
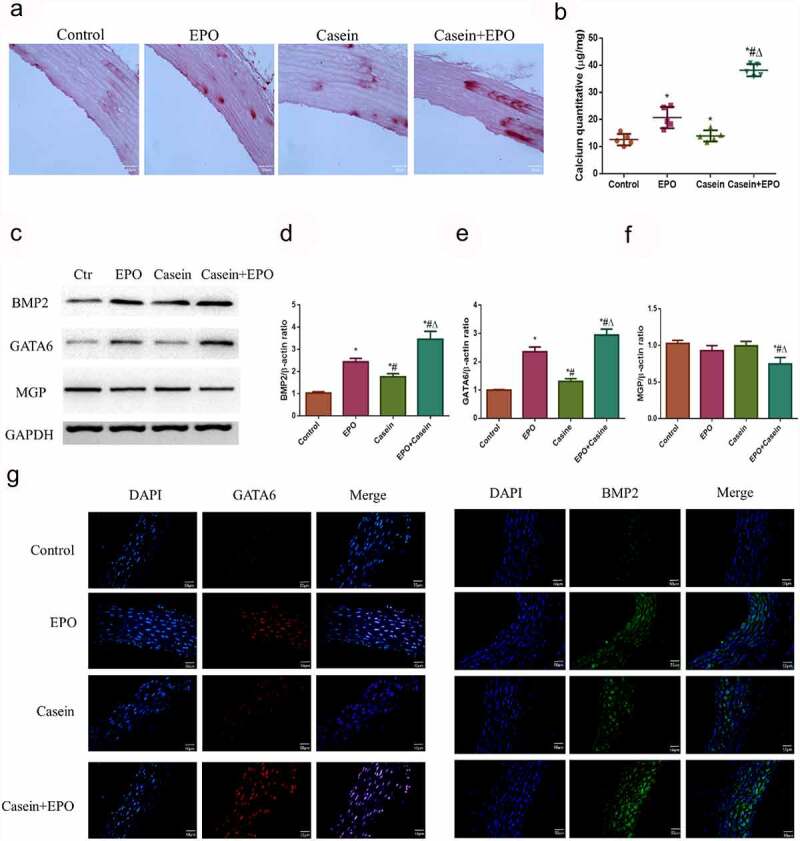
Chronic inflammation aggravated vascular calcification caused by EPO in vivo. Rats were injected intraperitoneally with 2000 U/Kg/W of EPO three times per week or subcutaneously with 10% casein (1.2 g/kg), lasting 20 weeks. A. Alizarin red staining of slices of abdominal aorta (4 μm) and B. calcium deposits quantification and a graph with statistical analysis. C. Western blot results of BMP2, GATA6 and MGP protein expressions. D-F. Densitometric analysis of protein expression. G. Expressions of BMP-2 and GATA6 in the thoracic aorta via immunofluorescence. The results are presented as mean ± SD; *n*=3. * p<0.05 vs. control group, # p<0.05 vs. EPO group, Δ p<0.05 vs. Casein group.

The systematic inflammation of rats was confirmed above by detecting the inflammation related cytokines TNF-α, IL-6, IL-1β and IL-10. To further determine whether inflammation could aggravate EPO-induced calcification in vivo, alizarin red staining was performed to determine calcium deposition in abdominal aortas. A small quantity of calcium deposition was uncovered in the abdominal aorta of rats administered with EPO and calcification was markedly promoted following casein treatment, indicating that chronic inflammation could aggravate EPO-induced calcification in vivo (Figure 4(a–b)). Furthermore, the levels of BMP2, GATA6, and MGP were investigated through Western blot (Figure 4(c–f)) and immunofluorescence (Figure 4(g)). Although the expression trends of BMP2 and GATA6 in each group were consistent with that in vitro, no significant changes were discovered in MGP protein expression in the EPO and the casein group compared with the control, while significantly decreased in the EPO+casein group. These data indicated that chronic inflammation could aggravate vascular calcification induced by EPO in vivo.

### TNF-α aggravates calcification in VSMCs induced by EPO through activating p38

**Figure 5.**
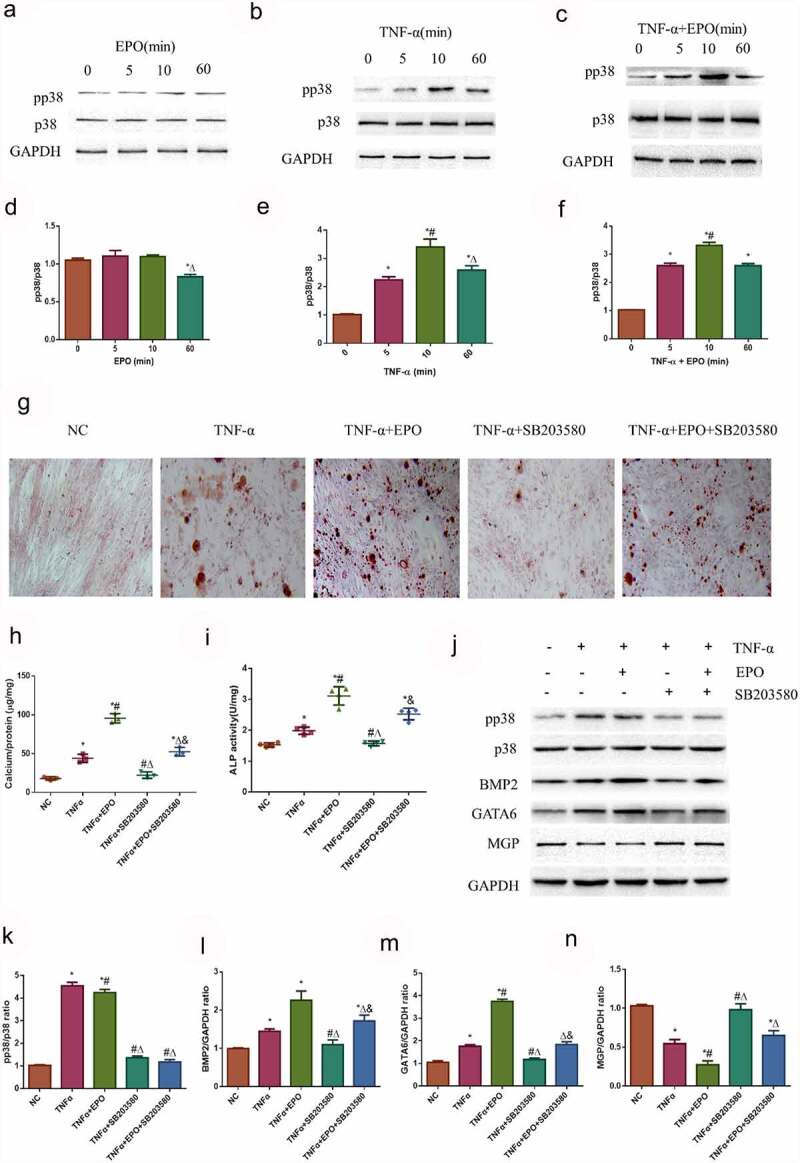
TNF-α aggravated calcification in VSMCs induced by EPO through activating p38 signal pathway. A-C. Western blot results of pp38 and p38. VSMCs were cultured with 250 U/mL single TNF-α, EPO or combined with 20 ng/mL TNF-α for different time. D-F. Densitometric analysis of protein expression, * p<0.05 vs. 0 min group, # p<0.05 vs. 5 min group, Δ p<0.05 vs. 10 min group. Then VSMCs were pretreated 10 μmol/L using SB203580 for 1 h and then treated with 20 ng/mL TNF-α in the presence or absence of EPO (250 U/mL). Calcium deposition was detected by G. Alizarin red staining (magnification ×200) H. Determination of calcium content I. ALP activity J. Western blot results of pp38, p38, BMP2, GATA6 and MGP K-N. Densitometric analysis of protein expression. The statistical results are expressed as mean ± SD; *n*=3. * p<0.05 vs. NC group, # p<0.05 vs. TNF-α group, Δ p<0.05 vs. TNF-α+EPO group, & p<0.05 vs. TNF-α+SB203580 group.

Whether the signaling pathway of TNF-α aggravated calcification in VSMCs induced by EPO was investigated. The MAPK (p38) pathway has been recognized as a principal downstream effector of pro-calcification factors. The MAPK (p38) pathway was examined to determine the mechanism of TNF-α-accelerated EPO-induced calcification. We found that there were no significant changes with EPO treatment in VSMCs as time passed (Figure 5(a,d)). However, when being treated with TNF-α as well as TNF-α combined with EPO, p38 increased in a time-dependent manner, reached the peak at 10 min, and decreased at 60 min (Figure 5(b, c, e, and f)). Effects of SB203580, an inhibitor of p38, was further investigated on calcification accelerated by TNF-α using alizarin red staining. SB203580 significantly inhibited calcification induced by single TNF-α and EPO+TNF-α combination in VSMCs at day 14, however calcium deposition was still observed in the TNF-α+ EPO+SB203580 group (Figure 5(g, h)). Also, pp38/p38 expression was significantly inhibited in TNF-α+ SB203580 and TNF-α+ EPO+SB203580 group (Figure 5(j, k)). Besides, SB203580 strongly inhibited ALP activity, protein expressions of BMP2 and GATA6 increased by TNF-α (Figure 5(i–m)). The protein expression of MGP was significantly increased by adding SB203580 (Figure 5(j, n)). These data suggested that TNF-α aggravated calcification in VSMCs induced by EPO through activating the p38 signaling pathway.

### ROS is involved in calcification induced by EPO and TNF-α

**Figure 6.**
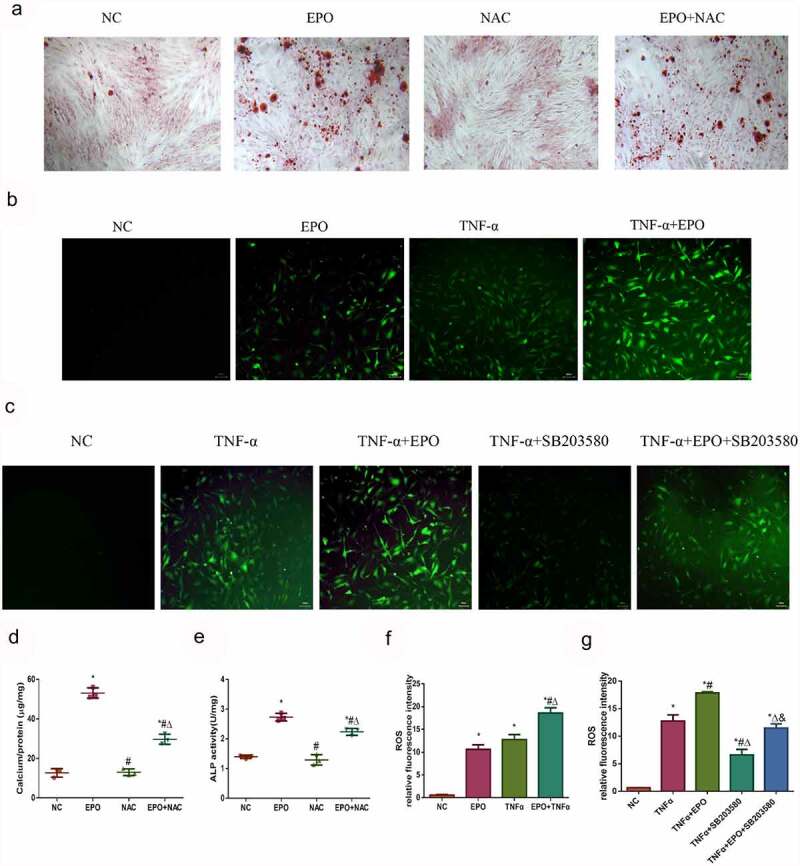
ROS was involved in calcification induced by EPO and TNF-α. A. Alizarin red staining (magnification ×200). VSMCs were cultured with EPO (250 U/mL) for 14 days with or without pretreatment of NAC (150 μmol/L). B–C. Typical immunofluorescence images of ROS in VSMCs (magnification ×200). Cells were treated with single 250 U/mL EPO or combined with 20 ng/mL TNF-α and with or without pretreatment of p38 inhibitor SB203580 (10 μmol/L) for 8 d. Determination of intracellular ROS levels employed fluorescent probe DCFH-DA. D. Determination of calcium content. E. ALP activity detection. * p<0.05 vs. NC group, # p<0.05 vs. EPO group, Δ p<0.05 vs. NAC group. F-G. ROS relative fluorescence intensity. The results are expressed as mean ± SD; *n*=3. * p<0.05 vs. NC group, # p<0.05 vs. EPO group, Δ p<0.05 vs. TNF-α group.

As reactive oxidative stress can affect calcification, levels of cellular ROS was detected using a DCFH-DA probe to figure out whether ROS mediated EPO and TNF-α induced calcification in VSMCs. Firstly, we used ROS scavenger NAC to demonstrate the effect of ROS on EPO-induced calcification. The results of alizarin red staining showed that NAC markedly decreased calcium deposition and ALP activity induced by EPO (Figure 6(a, d,e)), indicating ROS mediated calcification induced by EPO. The relative immunofluorescence intensities showed that EPO and TNF-α notably increased ROS compared with the control, and when the two worked together the ROS further increased (Figure 6(b,f)). Moreover, SB203580 decreased ROS induced by TNF-α (Figure 6(c,g)), arguing that TNF-α-induced p38 initiation also brought about ROS in VSMCs, which led to calcification.

## Discussion

Inflammation has been recognized as one of the risk factors of vascular calcification [31]. CKD patients suffer from a long-term micro-inflammation. Our previous study has demonstrated that the high-dose EPO can induce calcium deposition in VSMCs, while the mechanism remains to be fully explored [16]. It is unclear whether inflammation can play an additive or synergistic effect in EPO-induced vascular calcification. To investigate the regulation mechanism and effect of EPO-induced vascular calcification in the state of inflammation, we performed experiments in vivo and vitro. A rat model of systemic inflammatory state was constructed and stimulated with high-dose EPO to simulate the coexistence of high-dose EPO and inflammation in the clinic. High-dose EPO injection was performed referring to the descriptions of previous study [16,32]. We have constructed casein injection-induced model of chronic inflammation, and the increased multiple cytokine levels have been observed in the serum, which is more likely to mimic the chronic systemic inflammatory state in patients with inflammatory stress [33]. The oxidative cascade triggered by inflammatory cell activation in the arterial wall and the release of inflammatory factors can initiate the programmed osteogenesis process, including the central link of vascular calcification-the osteogenic phenotypic transformation of VSMCs, which ultimately leads to and even accelerates blood vessel calcification [34].

Numerous studies have reported that TNF-α is a typical inflammatory cytokine, which can promote the calcification of valve interstitial cells and VSMCs [35–37]. Csiszar et al. have proved that the common stimuli such as TNF-α, high intravascular pressure, hypertension, diabetes, and metabolic syndrome upregulate BMP2 expression in endothelial cells [38]. In agreement with previous studies, the experimental results of this study indicated that TNF-α-induced calcium salt deposition in VSMCs in vitro and up-regulated the expressions of BMP2 and GATA6. Moreover, simultaneous stimulation with EPO can achieve superimposed pro-calcification effects. However, Chang et al. in 2020 explored the ameliorative effects of EPO against vascular calcification in rats with chronic kidney disease [39]. In the systemic inflammation model stimulated by high-dose EPO, our results indicated that except TNF-α, inflammatory cytokines of serum IL-6 and IL-1β were significantly elevated compared with EPO or casein treated alone. Moreover, high-dose EPO combined with casein could lead to a small amount of calcium deposition on the slices of abdominal aorta compared with high-dose EPO or casein treated alone, significantly up-regulating the expressions of BMP2 and GATA6 compared with high-dose EPO or casein treated alone. The results revealed that the respective pro-calcification effects of EPO and inflammation had a superimposed effect, which significantly enhanced the effect of EPO on vascular calcification in rats under chronic inflammation. The specific mechanism might be related to the damage of the anti-calcification mechanism in vivo. Collectively, it is suggested that in the clinical prevention and treatment of CKD vascular calcification, the dose of EPO and the level of inflammation in patients should be controlled at the same time.

Previous studies have found that MGP functions as a key Gla-dependent inhibitor of bone morphogenetic proteins 2 and 4. Yao et al. have proved that interleukin 6 (IL-6) can increase the expression and secretion of heat shock protein 70 (HSP 70) in diabetic vascular diseases. HSP 70 served as an antagonist of endogenous MGP and is highly expressed in calcified atherosclerotic plaques [40]. Therefore, the inflammatory signal provided by IL-6 can enhance the effect of BMP2 and BMP4 in blood vessels by inducing HSP 70 to inhibit the effect of MGP. In this experiment, in vivo, high-dose EPO combined with casein could remarkably down-regulate the expression of MGP compared with control. Both EPO and inflammation could down-regulate the MGP protein in cells, which suggested that EPO and TNF-α might also be able to regulate the anti-calcification mechanism of VSMCs.

The results revealed that the MAPK (p38) pathway and ROS mediating in the process of TNF-α induced vascular calcification. Meanwhile we found that p38 can only be activated by TNF-α other than EPO, which has also been reported in mediating the oxidative and pro-inflammatory pathways [41]. And the p38 pathway has also been proved to be essential for calcification by various experiments. p38 pathway indicates the effects of regulating osteogenic differentiation, extracellular matrix deposition and mineralization after being activated by osteoinductive ligands such as BMP2, Wnt protein, and parathyroid hormone (PTH) in osteoblast cell lines or primary cells [42]. Taken together, it is concluded that in the process of inflammation and EPO combined to induce calcification, EPO does not depend on the activation of the p38 pathway, while inflammation aggravated vascular calcification by activating p38.

## Conclusions

In this study, experiments have demonstrated that high-dose EPO can lead to a small amount calcium deposition in rat abdominal aorta tissues and up-regulate osteogenesis-related proteins. The chronic inflammation can aggravate the effect of EPO in upregulating inflammatory factors TNF-α, IL-6 and IL-1β and in pro-calcification in vivo. In addition, high-dose EPO can induce calcification of VSMCs and increase intracellular ROS and ALP activities. The mechanism may be related to the activation of GATA6/BMP2 signaling axis by EPO that promotes the phenotype change of VSMCs, which in turn promotes calcification of VSMCs. And we also found that TNF-α exacerbates the calcification-promoting effect of EPO by activating p38 signaling.

## Highlights

1. High-dose EPO induces VSMCs calcification and increases intracellular ROS activity.
2. TNF-α exacerbates pro-calcification effect of EPO through activating p38 signal.
3. EPO can cause calcium salt deposition in abdominal aorta tissues.
4. Inflammation can aggravate the pro-calcification effect of EPO in the body

## List of abbreviations

EPO: erythropoietin;

VSMCs: smooth muscle cells;

ALP: alkaline phosphatase;

ROS: reactive oxygen species;

DCFH-DA: 2’,7’-dichlorofluorescein diacetate;

MGP: matrix gla protein;

TNF-α: tumor necrosis factor-alpha;

CKD: chronic kidney disease;

GATA6: GATA Binding Protein 6;

BMP2: bone morphogenetic protein-2;

IL-6: interleukin-6;

IL-10: interleukin-10;

IL-1β: interleukin-1 beta;

BCA: bicinchoninic acid;

HCL: hydrochloric acid;

TBST: Tris-buffered saline with Tween 20;

DEPC: diethylpyrocarbonate;

HSP70: heat shock protein 70;

PTH: parathyroid hormone
